# Tobacco packaging strategies aimed at undermining graphic health warnings

**DOI:** 10.18332/tid/109756

**Published:** 2019-07-03

**Authors:** Ji-eun Hwang, Sung-il Cho

**Affiliations:** 1 Institute of Health and Environment, Seoul National University, Seoul, Republic of Korea; 2 Department of Public Health Science, Graduate School of Public Health, Seoul National University, Seoul, Republic of Korea

**Keywords:** graphic health warning label, packaging and labeling, tobacco industry

**Dear Editor,**

The advertising and promotion of tobacco products are particularly heavily regulated and so marketing strategies at the point-of-sale are the main channels that connect current and future customers^1^. For this reason, the tobacco industry has devoted much attention to packaging design, especially with regard to elements such as color, logo, and name. Consequently, cigarette packaging not only communicates information about the product but can also influence people’s perception of the product as relatively safe and less dangerous to their health^2^.

Warning phrases and pictorial warnings must be displayed on tobacco packaging to clarify the dangers associated with the use of tobacco and to reduce the product’s value. However, unless plain or standardized packaging that excludes all cigarette advertising elements is obligatory, the tobacco industry may apply various design elements aimed at rendering the cigarette products more attractive within the maximum allowable area of the cigarette packaging^3^.

In South Korea, mandatory graphic health warnings on cigarette packaging were implemented from 23 December 2016^4^. The warning must occupy a total area of 50% (30% pictorial warning, 20% text warning) on both the front and back of the cigarette pack, according to the relevant legislation.

According to the Enforcement Decree of the National Health Promotion Act, detailed guidelines are in place regarding the format of the pictorial warning: the space for the warning should be outlined at the top of the packaging, and the space should include a pictorial warning and a warning phrase within the square border, delineated by a 2 mm thick black line. The text used for the warning phrase should be printed in Gothic font. The pictorial warning must be presented in the colors stipulated by the Minister of Health and Welfare, and the color used in the warning picture should be in clear contrast to that of the rest of the packaging, and in a complementary color.

To investigate the issues pertaining to cigarette packaging, we conducted a search of online news articles related to new cigarette products that were published between 23 December 2016 (when the graphic health warning policy was implemented) and 30 August 2018. We found five news articles that included photographs of new cigarette products, as well as descriptions of product packaging and product information, including nicotine content and price.

Figure 1 (a) shows the AFRICA GOLA product, as it was reported on *ETNews* on 12 September 2017^5^. Regarding this product’s packaging, a reporter commented: ‘This product is named GOLA and carries the motif of the African gorilla, which appears in famous Hollywood movies, and is depicted as witty and curious’.

**Figure 1:**
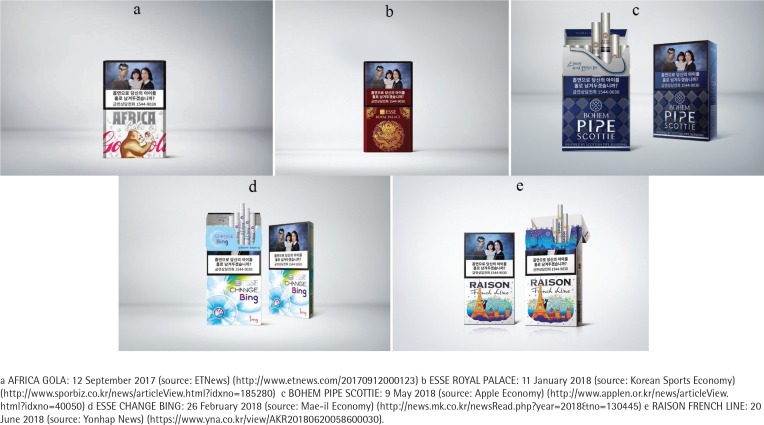
Cigarette packaging in South Korea

Figure 1 (b) shows ESSE ROYAL PALACE, as featured in the *Korean Sports Economy* on 11 January 2018^6^. The news reported that the product, which had hitherto been sold only in the Young-Honam area, was to be launched nationwide.

In addition, a reporter commented: ‘To emphasize the luxury of the product, the ESSE ROYAL PALACE packaging includes the symbol of the king, with an engraving of the golden dragon pattern of the *gonryongpo*, which was a dragon robe of kings, on the front of the product, and the castle of Suwon Hwaseong on the back of the product’.

Figure 1 (c) shows a new product, BOHEM PIPE SCOTTIE, announced on 9 May 2018^7^. A reporter at the *Apple Economy* wrote of the product’s packaging: ‘BOHEM PIPE SCOTTIE applied a Scottish traditional tartan check on a dark blue background’.

Figure 1 (d) illustrates a new product, ESSE CHANCE BING, which was reported on 26 February 2018, in *Mae-il Economy* ^8^. A reporter wrote that the ESSE CHANGE BING product is characterized by a fresh, cool look that differs from the existing ESSE CHANGE. The report explained that the packaging uses a radial graduation that spreads across the front and back, with a light blue background, to express the coolness. The symbol and logo were rendered in ultraviolet, which was the color of 2018 as selected by Pantone, an internationally renowned company that provides customized color standards^9^.

Figure 1 (e) shows a new product, RAISON FRENCH LINE, reported on 20 June 2018, by *Yonhap News* ^10^. The reporter observed that the cat that appears on the packaging, as the symbol of RAISON, demonstrates continuity with existing RAISON products, with the Eiffel Tower in Paris, France, added as a special motif.

A common feature of these new products is their aesthetically pleasing packaging. Furthermore, the color used in the warning phrase text was in the same color scheme as that used for the cigarette packaging, violating the relevant legislation that states that the warning phrase must be clearly displayed, in sharp contrast to the color scheme of the rest of the packaging. According to the National Health Promotion Act, the tobacco industry’s use of pictorial warnings and phrases that are in violation of applicable regulation is liable to punishment by imprisonment for up to one year or a fine of up to 10 million won (around US$9000). However, no penalty has hitherto been imposed for such a violation.

To maximize visibility and readability, the background color of the warning phrase should be complementary and in contrast to that used for the rest of the packaging. For this reason, the WHO Framework Convention on Tobacco Control (FCTC) guidelines also specify the use of complementary colors^11^. However, tobacco companies have persisted in using identical colors for the background of the warning phrase and the rest of the packaging, thereby reducing the text’s readability. Consequently, these packaging designs may undermine the Act’s objectives, by reducing the efficacy of the graphic health warning. If tobacco companies are not monitored with regard to their implementation of the policy, they are liable to engage in criminal or illegal practices. Therefore, a system should be established to monitor the activities of tobacco companies. It is also necessary to establish a pre-reporting system for assessment prior to a product’s launch, to verify that the packaging satisfactorily conforms to the guidelines.

## CONFLICTS OF INTEREST

The authors have completed and submitted the ICMJE Form for Disclosure of Potential Conflicts of Interest and none was reported.
